# Supplementary material for: Giant anisotropic magnetoresistance and nonvolatile memory in canted antiferromagnet Sr2IrO4
Source: Nat Commun. 2019 May 23;10:2280. doi: 10.1038/s41467-019-10299-6 (PMC6533248; doi:10.1038/s41467-019-10299-6)
Supplement: Supplementary file 1 — Supplementary Information [file 41467_2019_10299_MOESM1_ESM.pdf]

Supplementary Information

Giant anisotropic magnetoresistance and nonvolatile memory in canted antiferromagnet Sr_2IrO_4

H. W. Wang[1], C. L. Lu[1], J. Chen[2], Y. Liu[3], S. L. Yuan[1], S.-W. Cheong[4], S. Dong[2], and J. –M. Liu[5,6]

[1] *School of Physics & Wuhan National High Magnetic Field Center, Huazhong University of Science and Technology, Wuhan 430074, China*

[2] *School of Physics, Southeast University, Nanjing 211189, China*

[3] *School of Physics and Technology, Wuhan University, Wuhan 430072, China*

[4] *Rutgers Center for Emergent Materials and Department of Physics and Astronomy, Rutgers University, Piscataway, New Jersey, 08854, USA.*

[5] *Laboratory of Solid State Microstructures and Innovation Center of Advanced Microstructures, Nanjing University, Nanjing 210093, China*

[6] *Institute for Advanced Materials, Hubei Normal University, Huangshi 435001, China*

Supplementary Note 1: Anisotropic magnetotransport in J_{eff}=1/2 antiferromgnet Sr_2IrO_4

Sr_2IrO_4 has a layered antiferromagnetic (AFM) ground state, which has been demonstrated by various techniques, including resonant x-ray scattering and neutron scattering [1-3]. As sketched in Supplementary Fig. 1, the J_{eff}=1/2 moments (red arrows) are antiferromagnetically arranged in each IrO_2 layer, and show uniform canting angle relative to the *c*-axis. Because of canting, each IrO_2 layer has a net magnetic moment (green arrows) which is alternatively aligned along the *c*-axis. As a consequence, Sr_2IrO_4 is fully compensated at the ground state without showing macroscopic magnetization. With applying **H** above a critical value $\mathbf{H}_{flop}$, a flop transition is triggered, and then the net moments of IrO_2 layers are ferromagnetically coupled along the *c*-axis, leading to a weak ferromagnetic phase. The flop transition has been demonstrated using resonant x-ray scattering previously [1].

Accompanying with the flop transition (also known as AFM to weak FM transition), the resistance (*R*) shows a sudden drop, causing evident magnetoresistance (MR) in the samples. As shown in Supplementary Fig. 1b and c, the observed $R_c(\mathbf{H})$ trace correlates very well with $\mathbf{M}(\mathbf{H})$, suggesting that the field induced variation in magnetic order determines the magnetotransport in Sr_2IrO_4. This is in agreement with the previous results [4,5]. If having a look at the arrangement of the net magnetic moments (green arrows), one would see that the flop transition highly resembles the operation in a giant-magnetoresistance (GMR) device. Therefore, the large MR related to the flop transition is called as an atomic scale GMR-like effect in our work.

Achieving a large MR effect in antiferromagnets has been a focused issue for a long time. This has been intensively discussed for AFM heterostructures/multilayers, i.e. AFM/ non-magnetic-spacer/AFM. The existence of AFM counterpart to the GMR, called AFM-GMR, was theoretically proposed in such AFM structures [6,7]. The AFM-GMR was found to be purely an interface effect, and a spin-polarized state at the interface is essential [8]. For instance, a lower resistance will be achieved when the facing layers of antiferromagnets have the same spin orientation.

However, an experimental realization of the proposed AFM-GMR has encountered great challenge, since a theoretically assumed perfect interface quality can be an issue for epitaxial growth for artificial AFM-multilayers or heterostructures. For the Sr_2IrO_4 single crystal, it is a

natural antiferromagnet without such interface problem, and more importantly it hosts a layered AFM structure akin to the AFM multilayers: the AFM IrO_2 layers separated by non-magnetic SrO layers, and each IrO_2 layer has net magnetic moment μ_{net} due to the $J_{eff} = 1/2$ canting.

Indeed, a large MR related to the transition from AFM state to weak FM state is identified in Sr_2IrO_4. Very recently, the first principles calculations on the MR in Sr_2IrO_4 confirmed the critical role of scattering effects at domain walls [9]. This scenario is consistent with the scenario proposed in AFM multilayers.

To trigger the spin flop, a larger **H** is needed if it is aligned along the [110] direction than that along the [100] direction, because of the in-plane magnetic anisotropy. This gives rise to a $\Delta R = (R_{\mathbf{H}//[110]} - R_{\mathbf{H}//[100]})$ as **H** varies in a range [0.1 T to 0.25 T] shown in Supplementary Fig. 1b and c. The existence of a finite ΔR is vital for obtaining the large AMR effect (AMR = ΔR / $R_{\mathbf{H}//[100]}$). As clearly pointed out by Kim *et al.* [10], high quality Sr_2IrO_4 crystals are required to obtain evident and large in-plane anisotropy. In our experiments, a ΔR ~38 kΩ in our single crystals is obtained at T=35 K (where the large AMR of ~160% is identified) and it is a value larger than most reported values in literature, including those in semiconductors and tunnel junctions which usually show relatively large ΔR and AMR [11-13]. Another central physical ingredient for obtaining large AMR is to have a small $R_{\mathbf{H}//[100]}$. Our Sr_2IrO_4 single crystal sample along the *c*-axis is actually a natural heterostructure for spin-dependent transport, which offers high interfacial quality usually unavailable in artificial heterostructures. It implies that Sr_2IrO_4 single crystal would have very small $R_{\mathbf{H}//[100]}$. It also means that the larger the AMR as the larger the MR.

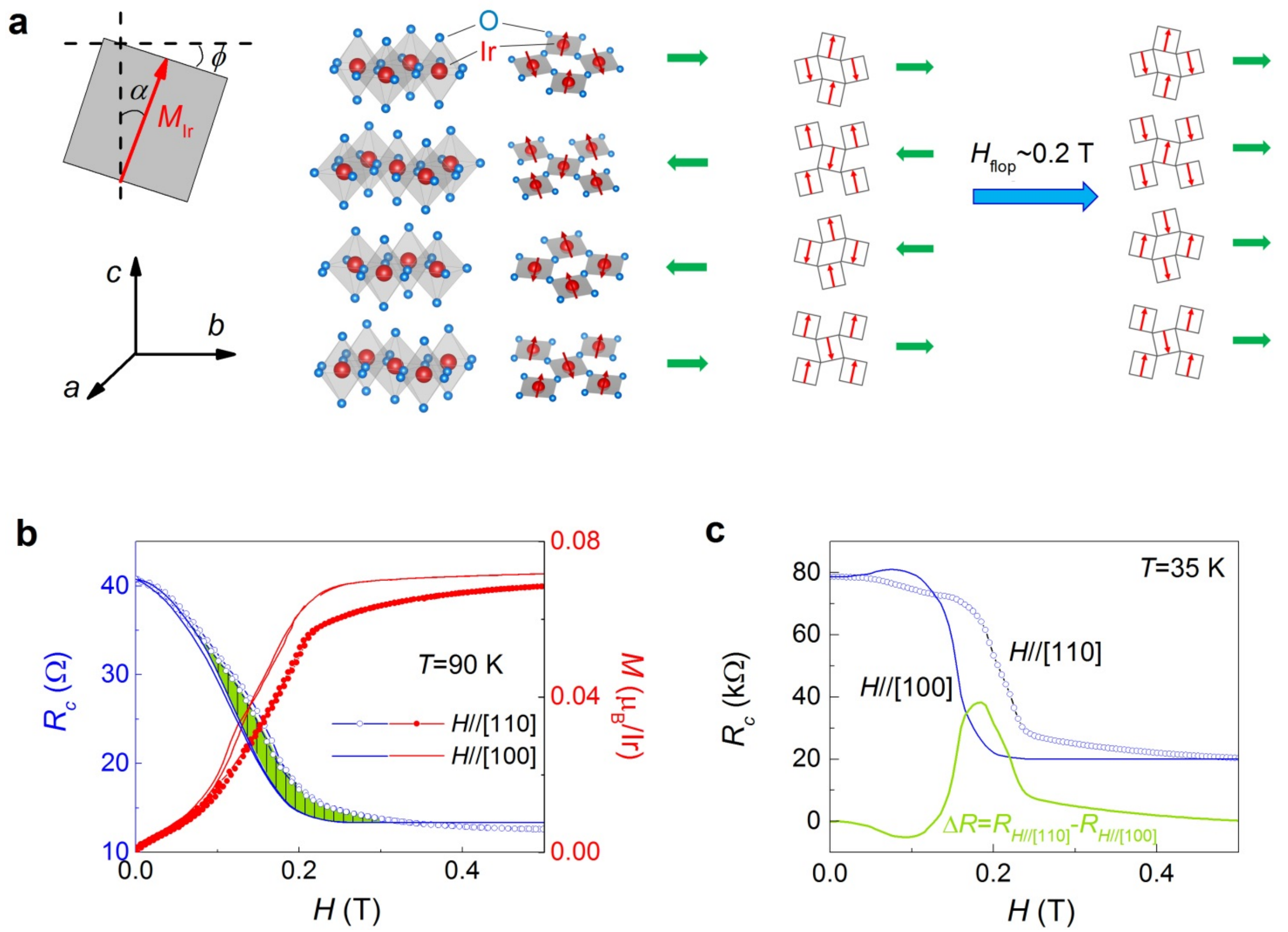

Supplementary Figure 1. Antiferromagnetism and physical properties in Sr_2IrO_4. (a) Sketch of the layered antiferromagnetic structure in Sr_2IrO_4. Blue and red spheres represent O and Ir, respectively. Red arrows indicate Ir magnetic moments, and green arrows mean the net magnetic moments of IrO_2 layers. An antiferromagnetic to weak ferromagnetic transition, i.e. the J_{eff}=1/2 moment flop transition, can be induced by $\mathbf{H}\sim \mathbf{H}_{flop}$ in Sr_2IrO_4. (b) Both **M** and R_c show remarkable variation at $\mathbf{H}_{flop}$. **M** and R_c were measured under various conditions at T=90 K. Because of in-plane magnetic anisotropy, R_c(**H**) shows evident anisotropy as **H** is applied along the [100] and the [110] directions, indicated by shadow. (c) Anisotropic magnetotransport measured at T=35 K. $\Delta R=R_{\mathbf{H}//[110]}- R_{\mathbf{H}//[100]}$ is found to be as large as ~38 kΩ.

Supplementary Note 2: Basic properties of Sr_2IrO_4 and $Sr_2Ir_{0.99}Ga_{0.01}O_4$

The crystals were crashed thoroughly and then powder X-ray diffraction (XRD) measurements were performed at room temperature. As shown in Supplementary Fig. 2, the refinements are high quality with small difference between the measured and refined spectra. The reliability parameter R_{wp} is 3.98% for Sr_2IrO_4 and 6.68% for $Sr_2Ir_{0.99}Ga_{0.01}O_4$. For Sr_2IrO_4, derived lattice parameters are a=5.493 Å and c=25.803 Å, which are in good agreement with literatures [3,14]. For $Sr_2Ir_{0.99}Ga_{0.01}O_4$, the lattice parameters are a=5.494 Å and c=25.792 Å, which are slightly different from the parent compound Sr_2IrO_4. This is under expectation, considering the close ionic radius between Ga^{3+} (0.620 Å) and Ir^{4+} (0.625 Å) [15].

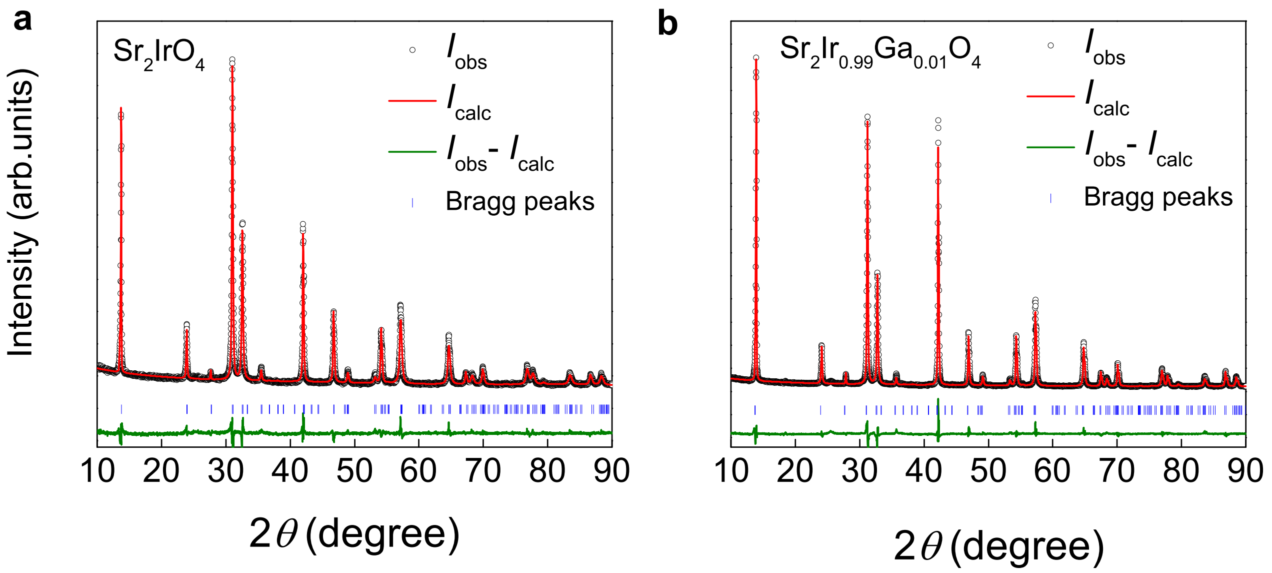

Supplementary Figure 2. **Structural characterizations.** Refinement of XRD patterns of (a) Sr_2IrO_4 and (b) $Sr_2Ir_{0.99}Ga_{0.01}O_4$.

Energy dispersive x-ray spectroscopy (EDX) equipped with a scanning electron microscope was used to check the elements' distribution and molar ratio in Sr_2IrO_4 and $Sr_2Ir_{0.99}Ga_{0.01}O_4$ crystals. All crystals are found to be homogeneous, according to our EDX results shown in Supplementary Fig. 3. The moral ratio of elements was estimated to be Sr:Ir ~2:1 in Sr_2IrO_4 crystals, and Sr:Ir:Ga~2:0.99:0.01 in $Sr_2Ir_{0.99}Ga_{0.01}O_4$ crystals. In $Sr_2Ir_{0.99}Ga_{0.01}O_4$, it is challenge to precisely determine the Ga-content because of the very low doping level (1%), and the Ga-content was estimated to be 0.9%~1.1%.

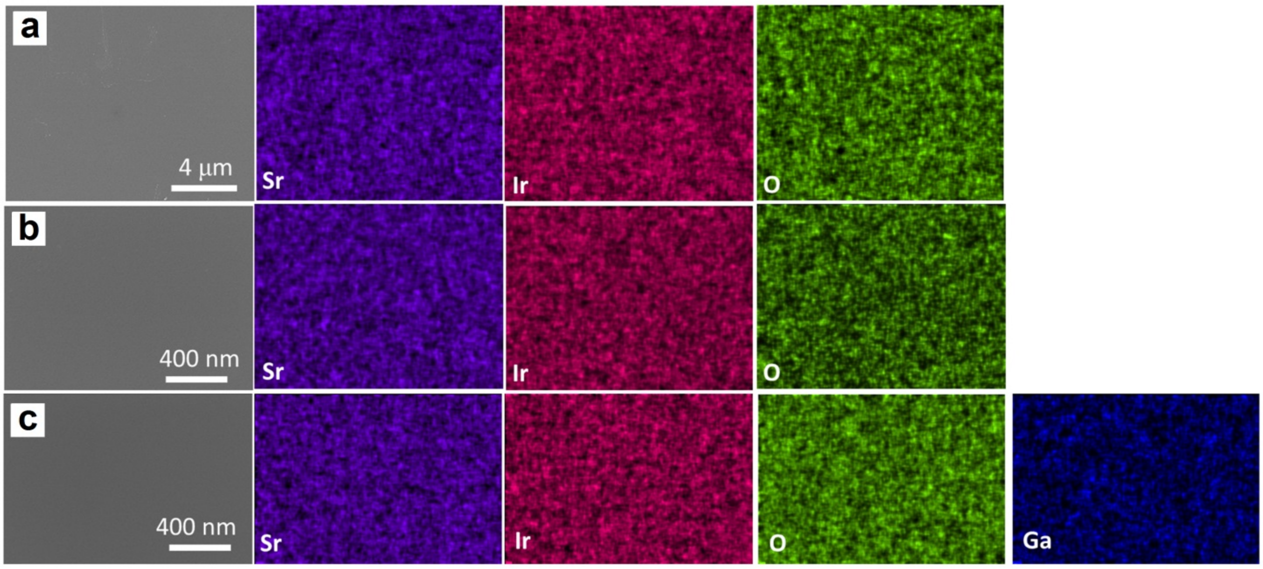

Supplementary Figure 3. **Microstructure characterizations.** (a) and (b) SEM images and corresponding elemental mappings of Sr, Ir, O for Sr_2IrO_4 crystal, which were obtained with different magnifications. (c) SEM image and corresponding elemental mappings of Sr, Ir, O, Ga for $Sr_2Ir_{0.99}Ga_{0.01}O_4$ crystal.

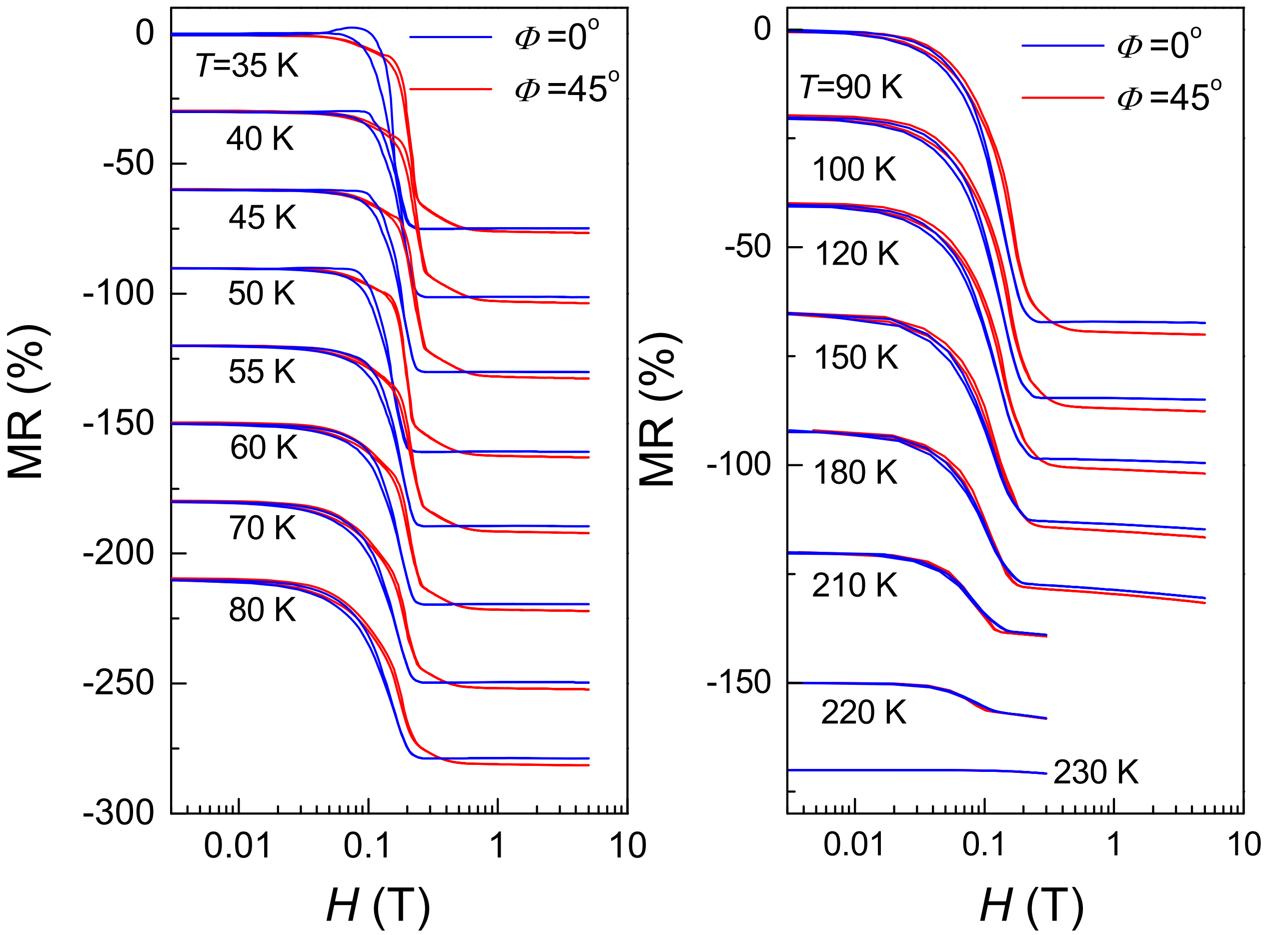

Supplementary Figure 4: Magnetotransport of Sr_2IrO_4 single crystal. Magnetoresistance data (MR=$R(\mathbf{H})/R(0)$-1) measured with **H** applied at Φ=45° (red curves) and Φ=0° (blue curves) at various temperatures. The MR curves have been shifted vertically to get a better view.

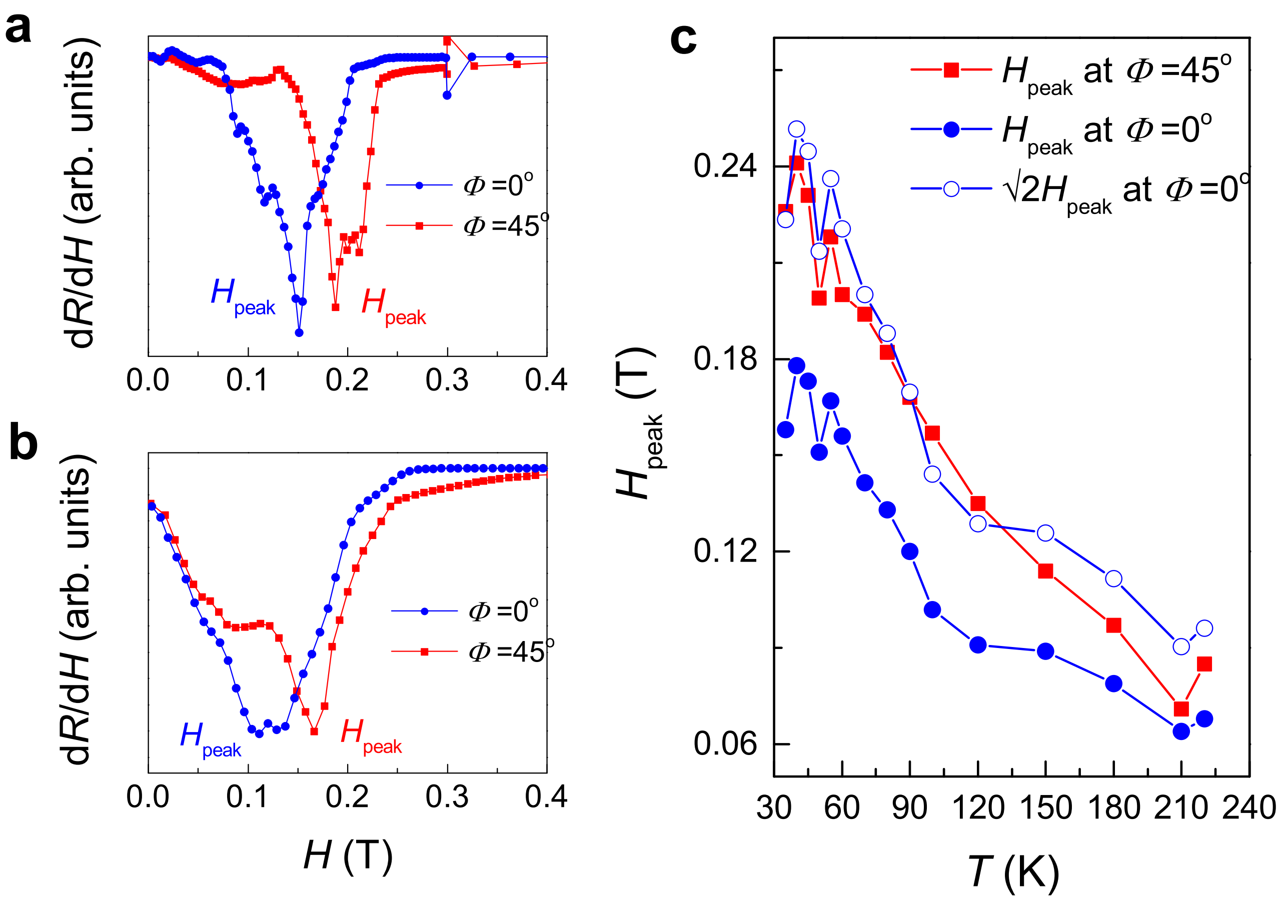

Supplementary Figure 5: Anisotropic magnetotransport of Sr_2IrO_4 single crystal. Differentiated $R(\mathbf{H})$ curves at (a) T=35 K, and (b) T=90 K. The critical fields of the MR drops are indicated with $\mathbf{H}_{peak}$. (c) Estimated critical fields $\mathbf{H}_{peak}$ as a function of T at Φ=45° (blue dots) and Φ=0° (red squares), which can be simply linked by a factor of √2 (blue circles).

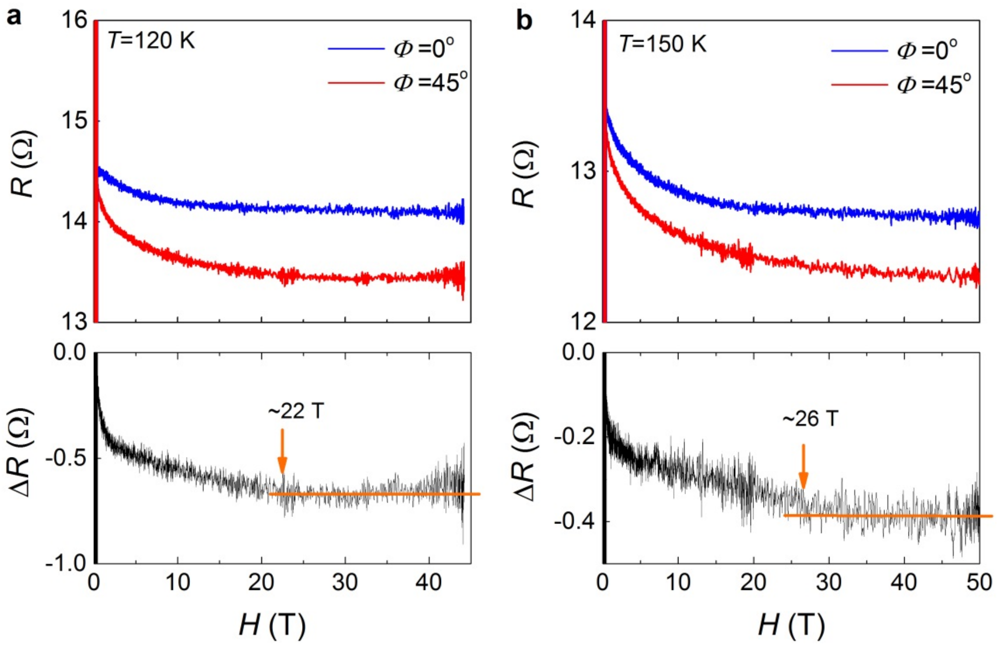

Supplementary Figure 6: **High magnetic field transport of Sr_2IrO_4.** High field *R*(**H**) curves measured at (a) *T*=120 K, and (b) *T*=150 K. It is seen that the anisotropic magneto-transport can persist up to ~50 T. The corresponding resistance discrepancy are shown at the bottom of (a) and (b), respectively. Roughly, at **H**>20 T, Δ*R* becomes a constant.

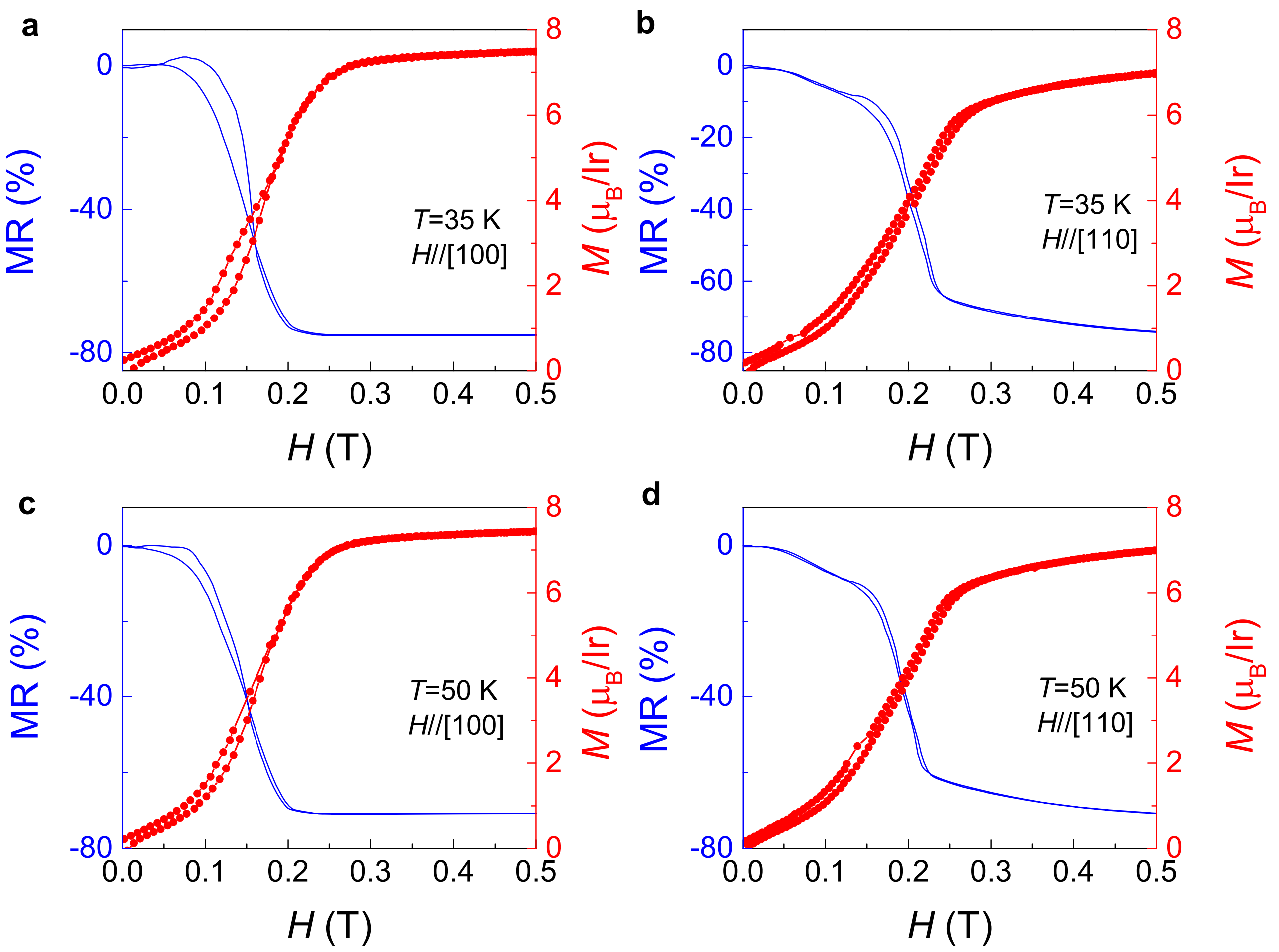

Supplementary Figure 7: Sr_2IrO_4 single crystal properties. Magnetoresistance and magnetization as a function of **H** measured at various temperatures: (a) *T*=35 K and **H**//[100], (b) *T*=35 K and **H**//[110], (c) *T*=50 K and **H**//[100], and (d) *T*=50 K and **H**//[110]. Clear hysteresis can be seen in both *R*(**H**) and **M**(**H**), which may due to the intensive domain wall motion in Sr_2IrO_4.

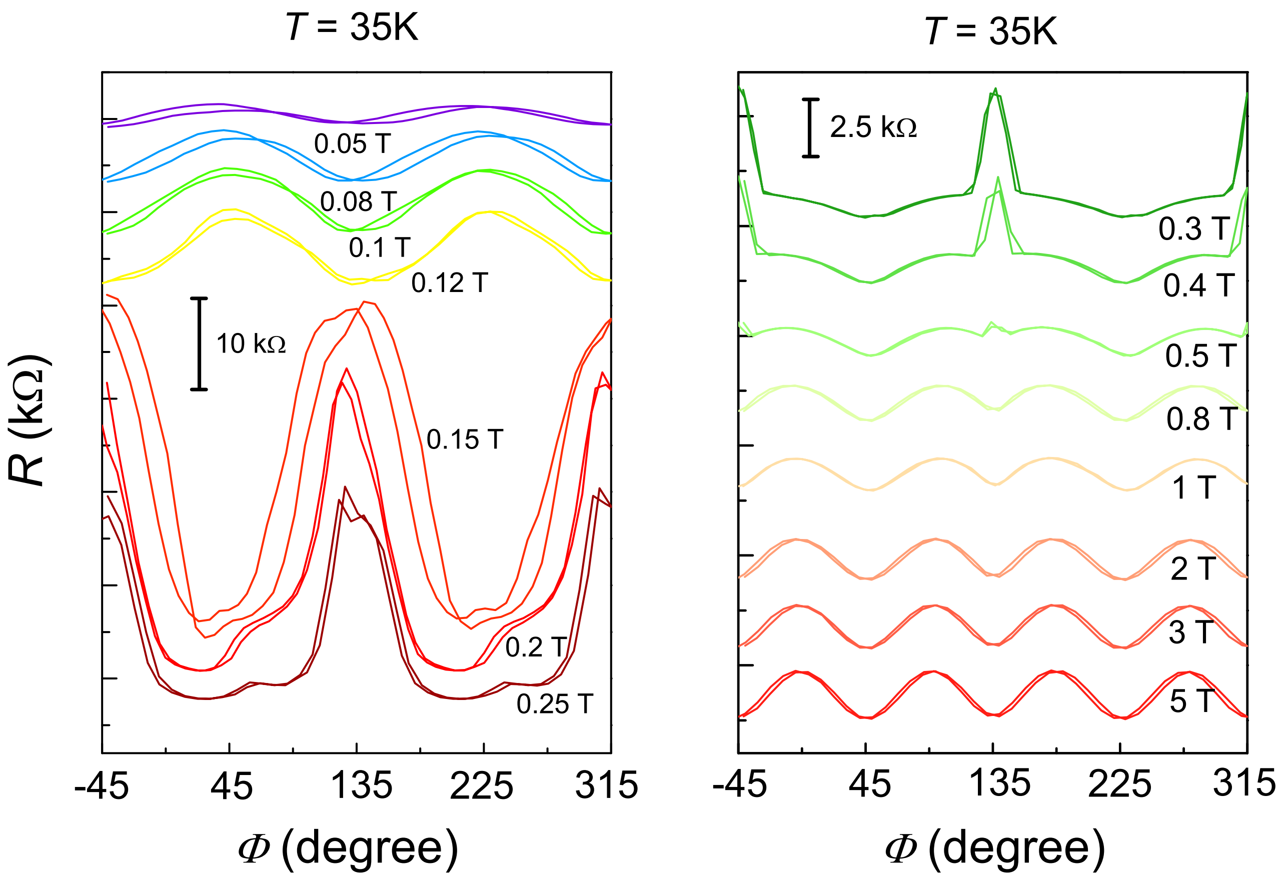

Supplementary Figure 8: Antiferromagnetic AMR at *T*=35 K. Anisotropic magnetoresistance measured at various magnetic fields at *T*=35 K. The measurements were performed by rotating **H** from -45° to 315°, and then back to -45°. The AMR curves have been shifted vertically to get a better view.

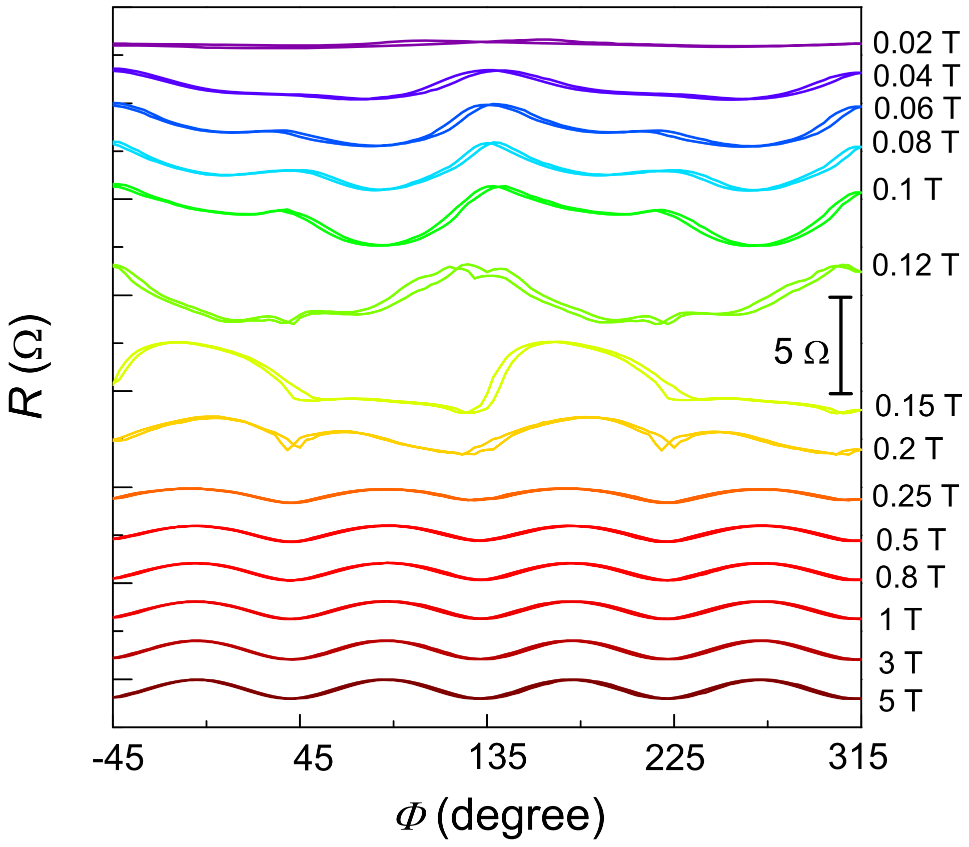

Supplementary Figure 9: Antiferromagnetic AMR at *T*=90 K. Anisotropic magnetoresistance measured at various magnetic fields at *T*=90 K. The measurements were performed by rotating **H** from -45° to 360°, and then back to -45°. The AMR curves have been shifted vertically to get a better view.

Supplementary Note 3: In-plane and out-of-plane magnetoresistance in Sr_2IrO_4

Recently, Lee *et al.* reported interesting AMR phenomenon in Sr_2IrO_4 [9]. An in-plane experimental set-up was used in their work, and a domain wall resistance scenario was proposed to explain the AMR phenomenon.

According to their calculations, the flop transition shows negligible effect on the electronic band structure in Sr_2IrO_4. Therefore, the large MR related to the flop transition is dominated by magnetic scattering, which is the common point between Lee's work and ours. The key difference between Lee's work and ours is sketched in Supplementary Fig. 10, i.e. the currents are in-plane in Lee's work but along the *c*-axis in our experiment. Therefore, the magnetic scattering at in-plane (IP) domain walls was considered by Lee *et al.*, while the magnetic scattering between IrO_2 layers was emphasized in our work. Although these two contributions for both in-plane and out-of-plane (OP) MR (i.e. $R_{ab}(\mathbf{H})$ and $R_c(\mathbf{H})$, respectively) coexist, MR experiments with different current orientations would lead different dominated mechanism.

Large IP- and OP-MR associated with the AFM - FM transition can be observed in Sr_2IrO_4, and the OP-MR effect (i.e. ~67% at T=90 K) is found to be much larger than the IP-MR (~40% at T=90 K), as show in Supplementary Figs 1 and 10. This is reminiscent of the observations in conventional GMR devices [16]. The IP- and OP-GMR effects have been studied for years, and the difference is ascribed to the different scaling lengths of the problem [16].

Here for Sr_2IrO_4, similar physics to the conventional GMR effects may be shared, while this topic definitely deserves for further investigations. We are not aware of any conclusive answer for this question, but three factors should be concerned. First, along the *c*-axis, the IrO_2 layers are separated by SrO layers, fundamentally different from the in-plane case. Therefore, the out-of-plane transport and magnetism is certainly different from the in-plane case. Second, the interlayer scattering takes place in every unit cell, which is much more popular than the domain wall reflection in the sample. Third, according to the calculations given of Lee *et al.*, complete and partial reflection of the electric current are expected at domain boundaries with 180°-rotation and 90°-rotation of μ_{net}, respectively [9]. For the interlayer scattering, only the 180°-rotation of μ_{net} exists. However, domain walls with 180°-rotation and 90°-rotation of the μ_{net} should be equally

distributed within the sample. These imply that the out-of-plane MR should be larger than the in-plane MR.

In addition, Wang *et al.* performed similar out-of-plane AMR measurements in Sr_2IrO_4 [5, 17], while a unique point contact technique was used. Indeed, very different results were obtained in comparison with ours. First, Wang *et al.* found that both MR and AMR strongly depends on the contact size (i.e. the AMR ratio was found to be increased from ~1% to 14% as the contact size was decreased at T=77 K). Second, a crossover from fourfold to twofold rotational symmetry of the AMR was identified in response to an increasing magnetic field. In our experiments, a standard method was used for the transport measurements, and no appreciable electrode-size (~1 mm^2) dependence was seen. Importantly, a fourfold AMR symmetry was eventually stabilized upon increasing magnetic field in our experiments, which was also observed by Lee *et al.* [9]. Therefore, the much smaller AMR magnitude (ten times smaller than ours) and the essentially different AMR symmetry revealed in Wang's work may arise from different physics related to the unique point-contact technique that was used in their experiments.

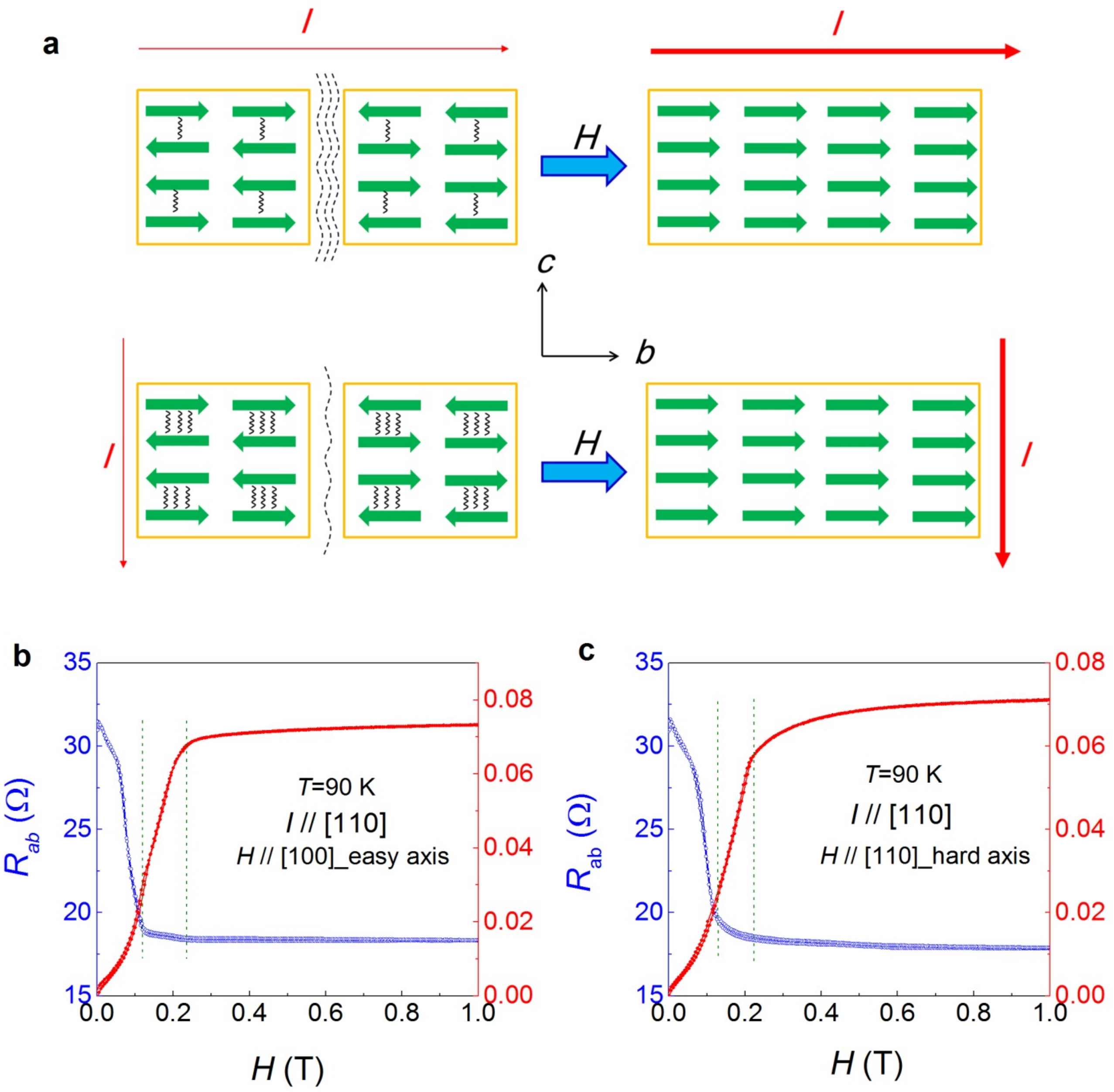

Supplementary Figure 10. Anisotropic magnetotransport and magnetism in Sr_2IrO_4. (a) Schematics of the two scenarios dominated by either domain wall resistance (electric current is applied within the basal plane) or a GMR-like effect (with electric current along the *c*-axis). For simplify, only the net magnetic moments (μ_{net}) of IrO_2 layers (green arrows) are shown. Electric current is indicated by red arrows. The scattering effects are indicated by wave lines. For *I*//*ab*-plane, the MR-related scattering is mostly contributed by domain walls, but for *I*//*c*-axis the interlayer scattering plays as a major role in the high resistance state. In-plane resistance R_{ab} and magnetization **M** as a function of **H** applied along: (b) the [100] direction, and (c) the [110] direction at T=90 K. The deviation between R_{ab}(**H**) and **M**(**H**) is indicated by olive dashed lines.

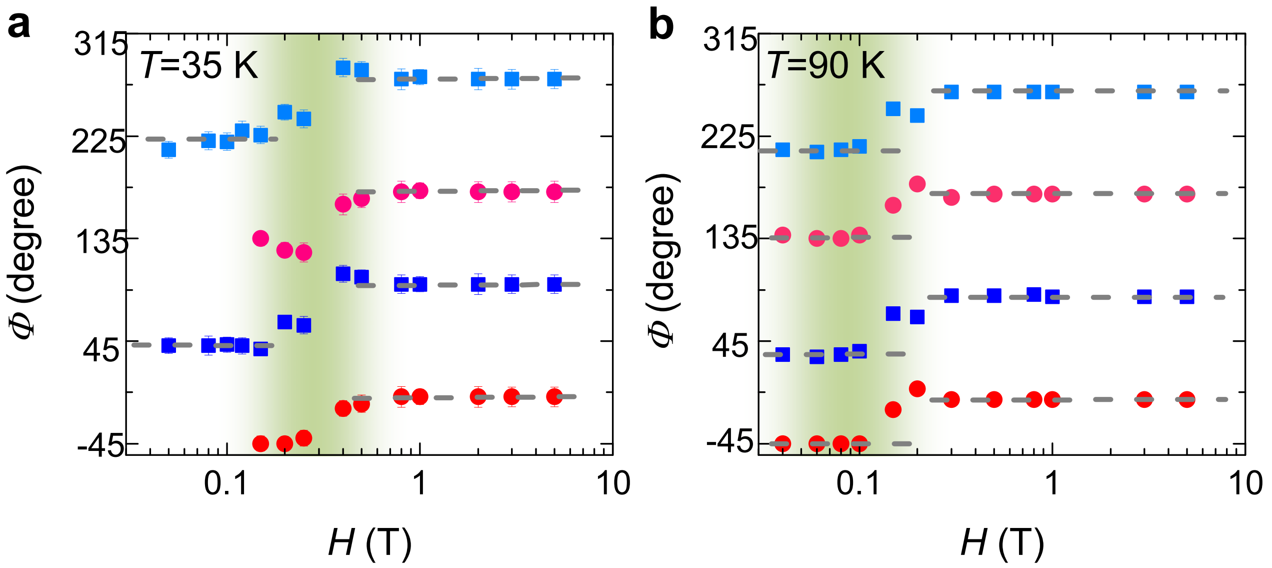

Supplementary Figure 11: **AMR reversal behavior.** Peak positions of the AMR traces as a function of **H** taken at (a) *T*=35 K, and (b) *T*=90 K. Three regions can be defined well, in agreement with the phase diagram shown in figure 1 in the main text. The shadowed region (olive) indicates the **H** range where the AMR is drastically enhanced.

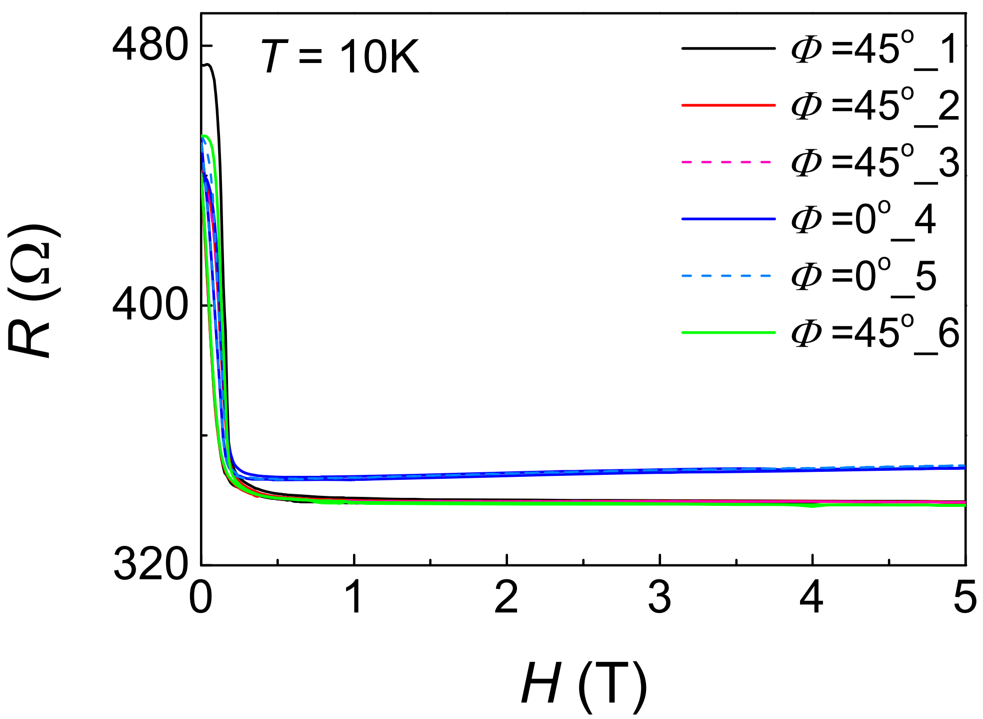

Supplementary Figure 12: **Dependence on writing magnetic field.** Resistance as a function of **H** measured at *T*=10 K for the sample with 1% Ga-doping. The measurements were performed by successive **H**-cyclings. For instance, *Φ*=0°_1 means the first **H**-cycle at *Φ*=0°. At high field region, higher conduction is evidenced at *Φ*=0°, which is the same as the non-doped sample Sr_2IrO_4.

Supplementary References:

[1] B. J. Kim *et al.*, Science 323, 1329 (2009).

[2] F. Ye *et al.*, Phys. Rev. B 87, 140406(R) (2013).

[3] C. Dhital *et al.*, Phys. Rev. B 87, 144405 (2013).

[4] M. Ge *et al.*, Phys. Rev. B 84, 100402 (2011).

[5] C. Wang *et al.*, Phys. Rev. X 4, 041034 (2014).

[6] J. Železný *et al.*, Nat. Phys. 14, 220 (2018).

[7] V. Baltz *et al.*, Rev. Mod. Phys. 90, 015005 (2018).

[8] P. M. Haney *et al.*, Phys. Rev. B 75, 174428 (2007).

[9] N. Lee *et al.*, Adv. Mater. 30, 1805564 (2018).

[10] J. Porras et al., arXiv: 1808.06920

[11] K. Y. Wang et al., Phys. Rev. B 72, 085201 (2005).

[12] J. Moser *et al.*, Phys. Rev. Lett. 99, 056601 (2007).

[13] B. G. Park *et al.*, Phys. Rev. Lett. 100, 087204 (2008).

[14] M. K. Crawford *et al.*, Phys. Rev. B 49, 9198 (1994).

[15] R. D. Shannon *et al.*, Acta Crystallogr. A 32, 751 (1976).

[16] A. Barthélémy *et al.*, J Magn. Magn. Mater. 242, 68 (2002).

[17] C. Wang *et al.*, J Appl. Phys. 117, 17A310 (2015).
